# In Vitro Drug Repurposing: Focus on Vasodilators

**DOI:** 10.3390/cells12040671

**Published:** 2023-02-20

**Authors:** Eduarda Ribeiro, Bárbara Costa, Francisco Vasques-Nóvoa, Nuno Vale

**Affiliations:** 1 OncoPharma Research Group, Center for Health Technology and Services Research (CINTESIS), Rua Doutor Plácido da Costa, 4200-450 Porto, Portugal; 2 CINTESIS@RISE, Faculty of Medicine, University of Porto, Alameda Professor Hernâni Monteiro, 4200-319 Porto, Portugal; 3 Institute of Biomedical Sciences Abel Salazar (ICBAS), University of Porto, Rua de Jorge Viterbo Ferreira 228, 4050-313 Porto, Portugal; 4 Cardiovascular R&D Center, Faculty of Medicine, University of Porto, Rua Doutor Plácido da Costa, 4200-450 Porto, Portugal; 5 Department of Surgery and Physiology, Faculty of Medicine, University of Porto, Rua Doutor Plácido da Costa, 4200-450 Porto, Portugal; 6 Department of Community Medicine, Information and Health Decision Sciences (MEDCIDS), Faculty of Medicine, University of Porto, Rua Doutor Plácido da Costa, 4200-450 Porto, Portugal

**Keywords:** vasodilators, repurposing drugs, in vitro

## Abstract

Drug repurposing aims to identify new therapeutic uses for drugs that have already been approved for other conditions. This approach can save time and resources compared to traditional drug development, as the safety and efficacy of the repurposed drug have already been established. In the context of cancer, drug repurposing can lead to the discovery of new treatments that can target specific cancer cell lines and improve patient outcomes. Vasodilators are a class of drugs that have been shown to have the potential to influence various types of cancer. These medications work by relaxing the smooth muscle of blood vessels, increasing blood flow to tumors, and improving the delivery of chemotherapy drugs. Additionally, vasodilators have been found to have antiproliferative and proapoptotic effects on cancer cells, making them a promising target for drug repurposing. Research on vasodilators for cancer treatment has already shown promising results in preclinical and clinical studies. However, additionally research is needed to fully understand the mechanisms of action of vasodilators in cancer and determine the optimal dosing and combination therapy for patients. In this review, we aim to explore the molecular mechanisms of action of vasodilators in cancer cell lines and the current state of research on their repurposing as a treatment option. With the goal of minimizing the effort and resources required for traditional drug development, we hope to shed light on the potential of vasodilators as a viable therapeutic strategy for cancer patients.

## 1. Introduction

**Figure 1:**
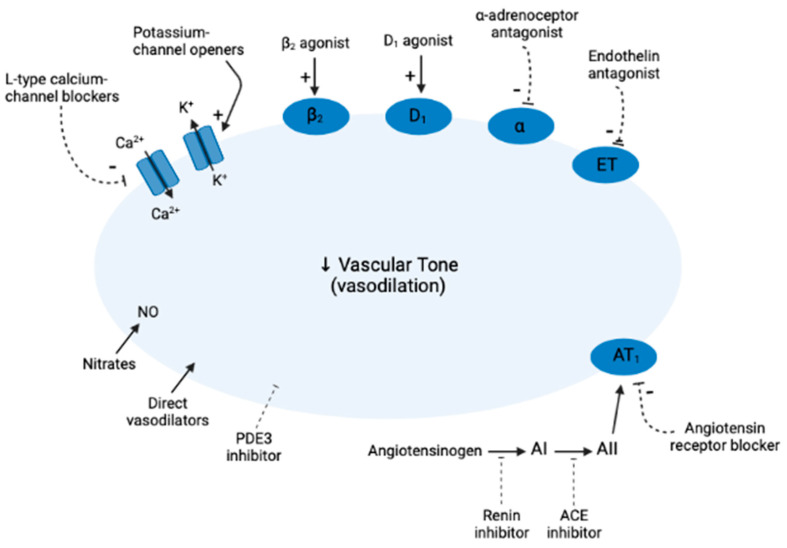
General mechanisms of action of vasodilators. Vasodilators may activate receptors that open potassium channels, produce hyperpolarization of the endothelium, and enhance calcium influx through receptor-operated calcium channels. Stimulation of β_2_ and D_1_ receptors causes smooth muscle relaxion, while α-adrenoceptor antagonists, endothelin antagonists, and angiotensin receptor blockers prevent the binding of norepinephrine, endothelin, and angiotensin II, respectively. Nitrates increase the amount of NO in vascular tone, causing vasodilation. PCE3 inhibitors prevent the phosphodiesterase enzymes from breaking down cAMP and cGMP in the cell, causing vasodilation and smooth muscle relaxation. The exact mechanism of direct vasodilators is still unknown.

Vasodilators encompass several classes of drugs that relax blood vessels. These drugs are used to treat cardiovascular problems such as hypertension, angina pectoris, and cardiac failure [1,2]. Vasodilators include medications that are categorized as angiotensin-converting enzyme inhibitors (ACEIs), angiotensin receptor blockers (ARBs), calcium channel blockers (CCBs), nitrates, direct vasodilators, calcium sensitizers, and phosphodiesterase 5 (PDE5) inhibitors (Figure 1). ACEIs are primarily used to treat hypertension and heart failure, preventing ACE-mediated conversion of angiotensin I to angiotensin II and the resulting increase in blood pressure. The reduction in plasma levels of angiotensin II by ACE inhibitors leads to increased plasma renin activity and decreased blood pressure, vasopressin secretion, sympathetic activation, and cell growth [3,4,5]. ARBs block the action of angiotensin II by selectively binding of angiotensin type 1 (AT1) receptors in tissues, which intermediates all the typical effects of angiotensin II such as vasoconstriction. CCBs promote vasodilation by reducing calcium flux into cells. These drugs are the most typically used in cardiovascular medicine for the treatment of not only hypertension but also angina and tachyarrhythmias [4,6]. CCBs or calcium antagonists promotes vasodilation (and reduce blood pressure) by reduction of calcium flux into cells. These drugs are the most usually used in cardiovascular medicine in the management not only of hypertension but also for tachyarrhythmias and angina [7]. The main roles of CCBs include dilatation of coronary and peripheral arterial vasculature, a negative inotropic action, reduction in heart rate, and slowing of atrioventricular conduction [8]. There are two classes of CCBs: dihydropyridines (DHPs), which have eminent selectivity for vascular smooth muscle cells than for cardiac myocytes, and non-DHPs, which have selectivity for cardiac myocytes and are used for treat cardiac arrhythmias [9,10,11]. Direct vasodilators are dilators of resistance vessels with little action on venous beds, used for severe refractory hypertension, malignant hypertension, and hypertensive emergencies [12]. Nitrate vasodilators embrace a group of organic nitrate esters that cause vasodilation by nitric oxide (NO) liberation [13,14], which can be used as monotherapy or add-on therapy in combination with other anti-angina drugs [15,16]. The molecular basis of nitrate pharmacotherapy is mediated through activation of endogenous NO-cGMP signaling pathways [17]. NO stimulates the soluble form of the enzyme guanylate cyclase in the smooth muscle cells of blood vessels. Guanylate cyclase produces cyclic guanosine monophosphate (cGMP) from guanosine triphosphate (GTP). cGMP in turn activates cyclic nucleotide-dependent protein kinase G which phosphorylates various proteins that play a role in decreasing intracellular calcium levels, leading to relaxation of the muscle cells and, thus, to dilation of blood vessels [14].

Calcium sensitizers are a recent class of inotropic drugs. They act on contractile proteins without increase intracellular calcium load, improving myocardial performance [18]. PDE5 inhibitors are commonly used in the management of erectile dysfunction [19].

All these characteristics and types of vasodilators represent ways they can affect cancer cells by increasing blood flow to the tumor, inhibiting the formation of new blood vessels, activating macrophages, or affecting several important signaling pathways. Therefore, despite the progressive research and recent advances in the development of effective drug treatments to treat solid tumors, the number of cases and deaths in cancer patients remains a major health problem. Drug repurposing aims to give to existing Food and Drug Administration-approved (FDA-approved) drugs new indications, rather than the original indication. Currently, the profile of drug reuse has essentially increased for three main reasons [20,21]. Firstly, the use of existing drugs for new purposes reduces drug development time by using what is already known about these drugs, including their pharmacodynamics, pharmacokinetics, uncommon and common toxicities, dosing scheme, and mechanism of action. This means, secondly, that most preclinical and Phase 1 (determine if the effects observed in animal tests also occur in humans) clinical development steps can be shortened. In this way, drug repurposing grants a significantly faster path to Phase 2 (determining therapeutic efficiency for the target disease) and Phase 3 (comparison of the new drug with existing and marketed drugs) trials compared to traditional drug discovery and development, where dosing, safety, and toxicity profiles are not fully known. Thirdly, as a result of the above, the financial investment related to development is substantially lower [22,23]. Moreover, these drugs, which have been used for other indications previously, already have reliable data on drug safety and are often inexpensive [23]. In additional to this, in silico drug repurposing has gained great prominence as a complement to in vitro and in vivo studies. These studies have the advantage of being quick to perform, low-cost, and able to reduce the use of animals in toxicity tests [24,25]. Treating cancer can be a complex and challenging task due to the many different types of cancer since each has its unique set of characteristics and behaviors. For example, some cancers are highly aggressive and fast-growing, while others are slow-growing and less aggressive. Some cancers respond well to traditional treatments such as chemotherapy and radiotherapy, while others do not [4,20]. Additionally, many cancers are characterized by genetic mutations or other abnormalities that make them resistant to treatment. One way to address the difficulties of treating different types of cancer is to focus on the mechanism of action of a given treatment. In other words, understanding how a particular treatment works at a molecular level can help identify which types of cancer are most likely to respond to that treatment [21,22]. Therefore, it is important to note that the effects of vasodilators on cancer cells vary depending on the specific drug and the type of cancer being studied (see Table 1). The effects of vasodilators on cancer cells in a laboratory setting may not necessarily translate to the same effects in humans. Further research is needed to fully understand vasodilators’ impact on cancer cells and determine their potential as a treatment for cancer. In the next section of this review article, we examine the literature on the effects of each class of vasodilators in various cell lines, highlighting how these drugs can exhibit both protumor and antitumor properties, depending on the specific drug and cancer type being studied.

## 2. Angiotensin-Converting Enzyme (ACE)

### 2.1. Enalapril

Enalapril is an active angiotensin-converting enzyme inhibitor, used in clinical practice for the treatment of several conditions, including heart failure and hypertension [148]. This substance can reduce cell proliferation and cause cell death, activating cell signaling pathways that involve TGF-β as the main regulator and tumor suppressor factor in epithelial cells [26,27,28]. Yang et al. showed that enalapril can be used as a chemotherapy sensitizer, strongly restoring chemotherapeutic sensitivity and improving the efficacy of 5-FU in colorectal cancer (CRC). In this study, through MTT assays, it was demonstrated that the use of enalapril in CRC cell lines had a limited effect even at high concentrations (100–2000 μM) for 72 h. However, the combination of enalapril with 5-FU extremely decreased cell viability in HCT116 and SW620 cell lines at 72 h. As tumor invasion, metastasis, and therapeutic resistance are closely related to epithelial–mesenchymal transition (EMT), Yang et al. assessed the effect of the combination use of these two drugs on EMT, finding that the cotreatment had a synergistic effect on suppressing EMT by upregulating the epithelial cell marker E-cadherin and downregulating the mesenchymal cell markers Vimentin and Snail, suggesting that inhibition of the EMT process may be considered as another possible mechanism via which enalapril potentiates the antitumor effect of 5-FU in CRC. The results of this study confirm that the synergistic effect between enalapril and 5-FU enhanced the chemosensitivity of CRC cells to 5-FU mainly through inhibition of cell proliferation [29]. Similar to this article, Mostafapour et al. exhibited the therapeutical impact of enalapril and its combination with 5-FU in the treatment of CRC. Studying three CRC cells (CT26, HT29, and SW40), they demonstrated that an increase in the enalapril concentration could lead to a decrease in cell growth, being more efficiently combined with 5-FU. Furthermore, the effects of enalapril on migratory behaviors have been investigated, and the results revealed a substantial reduction in the migration of CRC cells after 20 h [30]. Ozlem et al. examined, in the first phase of their study, the possible apoptotic and cytotoxic effects of enalapril in HL60 cells, as well as the potential mechanisms involved in cell death. Enalapril reduced the viability and proliferation of HL60 cells in a time- and dose-dependent manner, having an IC_50_ value of 7 μM. When they studied the possible apoptotic effect in these cells, the results showed that necrotic cells were in abundance compared to apoptotic cells. In the second phase of this study, Ozlem et al. intended to identify the pathway(s) involved in the cytotoxicity of enalapril in HL60 cells. Therefore, because STAT proteins play an important role in cellular processes and the dysregulation of the STAT pathway can lead to formation of malignant cells [31], they investigated the expression gene levels of STAT3, ATAT5, and STAT5B by qRT-PCR. The results showed that only changes in expression levels of STAT3 and STAT5A were statistically significant, whereas these proteins can play a significant role in enalapril-induced leukemia cell death [32].

### 2.2. Captopril

Captopril (D-3-mercapto-2-methylpropanoyl-L-proline) is an orally active competitive inhibitor of ACE, approved by the FDA in 1981 as an antihypertensive drug [33,149]. Captopril is prescribed in the management of congestive heart failure and hypertension [150,151,152]. Captopril has been described to inhibit mitosis in some cell types including canine renal epithelial cells [34], human neuroblastoma cells [35], human lung fibroblasts [36], hamster pancreatic carcinoma cells [37], and a cell line derived from a human salivary gland carcinoma [33]. Captopril was proven to be highly cytotoxic to HCT116 and DU145 cancer cells with IC_50_ values of 1.5 and 1.2 mg/mL. The cells, after 24 h of treatment, became granulated and rounded, dethatching the monolayer along with an increase in drug concentration. Captopril was also able to inhibit the migration of cancer cells in a concentration-dependent manner [38]. Another study was able to prove that, in the presence of sub-physiologic concentrations of CuCl_2_, captopril inhibits proliferation, thymidine incorporation, and mitochondrial dehydrogenase activity of Hs578T carcinoma cells in culture [33] Shen et al. demonstrated that captopril increases the antitumor effects of bevacizumab via inhibition of fibroblast contraction and extracellular matrix deposition, thereby reducing liver metastases stiffening [39]. Treatment of CRC with this vasodilator resulted in downregulation of Wnt/β-catenin signaling pathway [40]. Another study using captopril to deplete the overexpression of extracellular matrix (ECM) proved that this delivery significantly downregulated ECM by blocking the TCF-B1/Smad2 signaling pathway in pancreatic cancer [41]. In gliosarcoma cells, captopril decreased the expression of protein MMP-2 and, consequently, the migratory capacity [42].

### 2.3. Perindopril

Perindopril is used alone or in combination with other drugs to prevent heart attacks and to treat patients with high blood pressure. Perindopril can be highlighted in the group of ACE inhibitors due to its dose-dependent and long-lasting blood pressure-lowering effect, via the protection of blood vessels (improves endothelial function and decreases wall stiffness) and a decrease in variability of blood pressure [153,154]. Concentrations of VEGF mRNA and the expression of VEGF protein in human solid tumors correlate positively with malignant progression [155]. Yoshiji et al. reported that this ACE inhibitor considerably inhibited tumor growth and angiogenesis in hepatocellular carcinoma cells, along with suppression of the vascular endothelial growth factor (VEGF) level [156]. Another study demonstrated that 1 μM perindoprilat greatly inhibited VEGF expression, although it did not suppress the proliferation of KB cell line [157]. Zakaria et al. revealed that perindopril increased the antitumor effect of sorafenib via downregulation of EpCAM and leptin/Wnt/β-catenin pathway and overexpression of aldehyde dehydrogenase 1 (ALDH1) [158].

### 2.4. Trandolapril

Trandolapril is a nonsulfhydryl prodrug that has been used after myocardial infarction and in the treatment of congestive heart failure and hypertension [159]. Trandolapril was described to inhibit the cell growth of K562, KU812, and U937 cell lines at 1 mM and HL60 cells at 0.02 mM. Additionally, trandolapril induced cell apoptosis, increasing the percentage of apoptotic cells in the K562 cell line [43].

## 3. Angiotensin Receptor Blockers (ARBs)

### 3.1. Azilsartan

Azilsartan was the eighth approved ARB for the management of hypertension in February 2011 [160,161]. Azilsartan medoxomil (potassium salt) is a prodrug that, when hydrolyzed, liberates azilsartan, its active form [162]. Furthermore, azilsartan was shown to induce ROS production, cytochrome c release, and cytotoxicity in HepG2 and A549 cell lines [60,61]. In another study, azilsartan exhibited inhibition of cell growth, as well as induced apoptosis and cell-cycle arrest in breast cancer cell lines MCF-7 and MDA-MB-231. In addition, azilsartan treatment was shown to reduce the expression of NF-κB mRNA and IL-6, JAK2, and STAT3 proteins, thus promoting many cellular pathways in malignant tumors such as proliferation, metastasis, invasion, and angiogenesis, resulting in the suppression of the NF-κB/IL-6/JAK2/STAT3 signaling pathway in breast cancers [62].

### 3.2. Candesartan

Candesartan cilexetil is classified as an ester prodrug that, during absorption in the gastrointestinal tract, is converted to candesartan [163]. This vasodilator was approved by the FDA in June 1998 for the treatment of congestive heart failure and hypertension [164,165,166]. Candesartan is also used off-label to treat conditions including cerebrovascular accident or stroke, diabetic nephropathy, left-ventricular hypertrophy, and migraines [166]. A study reported that candesartan treatment inhibits the migratory behavior of CRC cells (CT-26 and SW480) via downregulation of MMP3/9 and induction of E-cadherin. Additionally, candesartan inhibits Wnt/β-catenin signaling via downregulation of cyclin D1, surviving, and MMP mRNA levels [63]. Recently, inhibition of the Wnt/β-catenin signaling pathway was shown to be related to suppressing tumor cell migration and metastasis [64], while the MMP enzymes were reported to be involved in cell migration and invasion [65,167]. Another study proved that candesartan combined with TRAIL, a member of the TNF family of cytokines that promotes apoptosis, was able to upregulate DR5, leading to apoptotic cell death in HCC-15 and A549 lung cancer cell lines. Candesartan had an effect on increasing autophagosome formation and caused defective lysosomal degradation. Furthermore, inhibition of AMPK phosphorylation via treatment with this vasodilator confirmed that defective autophagy triggers apoptosis by conferring cellular oxidative stress [66]. Another study proved that this vasodilator had an antitumor effect on breast cancer, enhancing liposome penetration and depletion of the tumor stroma [67]. Irbesartan inhibited the production of MCP-1 and the accumulation of CCR^2+^ inflammatory fibrocytes and monocytes in the inflamed colon, preventing the development of colitis-associated tumors [68].

### 3.3. Irbesartan

Irbesartan was the third ARB approved by the FDA; it is a potent and selective angiotensin II subtype 1 receptor antagonistic indicated for treatment of hypertension, including patients with type 2 diabetes mellitus and nephropathy [168,169]. A study demonstrated that irbesartan has the ability to inhibit metastasis by disrupting angiotensin II-induced adhesion of tumor cells to endothelial cells in hepatocellular carcinoma (HCCLM3, HMHCC97-H, HMHCC97-L, SMMC-7721, Huh-7, Hep-3B, and PLC) [69]. Another study revealed that irbesartan could suppress the cell proliferation effects of angiotensin II in breast cancer cells by inhibiting AT1R signaling [70].

### 3.4. Losartan

Losartan is as angiotensin AT1 receptor antagonist approved by FDA for the treatment of hypertension [170]. Zhao et al. exhibited that, through inhibition of the PI3K/AKT pathway, losartan could induce apoptosis in the MCF-7 cell line [71]. Another study, in agreement with the previous study, demonstrated that this vasodilator could induce apoptosis via inhibition of the PI3K/AKT pathway and expression of p53 and BAX levels in the CT26 cell line. Losartan administration increased MDA levels and reduced total thiol concentration and catalase activity, suggesting that changes in oxidant/antioxidant status may be one of the mechanisms underlying the antitumor activities of the drug losartan against colon cancer cells [72]. In another study, Takagi et al. provided evidence that losartan potently augmented the anticancer properties of lenvatinib against human liver cancer cell growth. The results showed that this ARB efficiently inhibited AT-II-stimulated cell growth and induced apoptosis in numerous human liver cancer cell lines [73]. It is known that AT-II can promote tumor growth in hepatocellular carcinoma (HCC) [74,75]. Losartan could reduce the ECM, leading to an increase in antitumor immunity, as exemplified by the reduction in tumor size and lung metastasis [76]. The combination of losartan with radiotherapy enhanced tumor control and inhibited metastasis of breast cancer via reducing tumor hypoxia [77]. On the other hand, cotreatment of losartan and chemotherapy enhanced the chemotherapy effects and decreased ascites in ovarian cancer [78]. This ARB inhibited cell growth and caused cell-cycle arrest at the G1 phase in CRC cell lines. Additionally, the tumor growth was reduced, and tumor cell necrosis was enhanced. Moreover, angiogenesis and metastasis were reduced via the inhibition of MM2 and MMP9 [72].

### 3.5. Olmesartan

Olmesartan is an oral inhibitor of angiotensin AT1 receptor, used to manage blood pressure and treat cardiovascular disease [171]. Kurikawa et al. described that olmesartan suppresses cell proliferation in hepatic cells [79]. Olmesartan inhibited cell growth in mice, being considered a good candidate in pancreas cancer treatment [80]. It has also been reported that olmesartan has cytotoxic activity against MCF-7 and HeLa cell lines [81,172]. Another study showed that olmesartan significantly increased intercellular ROS, which can be considered as a possible mechanism in olmesartan-induced toxicity in MCF-7 and HeLa cell lines, thus corroborating the documented information that an excessive amount of ROS can cause oxidative damage to proteins, DNA, and lipids and can lead to mitochondrial dysfunction, provoking oncogenic transformation, as well as increased metabolic activity, necrosis, and apoptosis [82,83,84]. The combination of telmisartan and docetaxel was shown to inhibit growth in tumor cells (PC-3, DU145, MDA-MB-468, and HEK cell lines) and reduce the expression levels of Snail and Slug genes [85].

### 3.6. Telmisartan

Telmisartan, a nonpeptide blocker of the angiotensin II AT1 receptor, is approved for the management of hypertension [173,174]. Telmisartan was shown to inhibit cell proliferation in a time- and dose-dependent manner in prostate cancer [175]. A study showed that telmisartan induced cell-cycle arrest at the G0/G1 phase in human GIST-T1 cells, via a reduction in the expression of cyclin D1 [86]. Another study showed that telmisartan inhibits cell growth by inducing apoptosis in many types of cancer, including gynecological [87] and urological [88]. On the other hand, two different studies showed that telmisartan inhibited cell proliferation through the induction of cell cycle arrest in cholangiocarcinoma cell lines [89] and human esophageal squamous cell carcinoma [90]. Cotreatment of telmisartan with ADH-1 resulted in a reduction in cell attachment to N-cadherin-coated plates on PC-3, MDA-MB-468, and DU145 cell lines. Another cotreatment of telmisartan with docetaxel reduced cell migration only in PC-3 and MDA-MB-468 cell lines [91]. Another study demonstrated that this ARB could inhibit proliferation in a dose- and time-dependent manner and induce cell-cycle arrest at the S phase in two glioma cell lines (U87 and U251) [92]. Mielczarek-Puta et al. demonstrated that the use of telmisartan in CCR influences the antiproliferative activity of linoleic acid [93]. Another study showed that telmisartan inhibited cell growth in a dose-dependent manner and caused cell-cycle arrest at the G0/G1 phase in gastric cancer [94].

### 3.7. Valsartan

Valsartan is a nonpeptide tetrazole that selectively inhibits angiotensin II AT1 used in the treatment of hypertension [176]. Wang’s research revealed that valsartan reduced proliferation, prevented invasion, and increased the radiation sensitivity of the CNE-2 cell line [95].

## 4. Calcium Channel Blockers (CCBs)

### 4.1. Amlodipine

Amlodipine, a DHP CCB, is normally used to cardiovascular diseases such as angina and hypertension [177]. Amlodipine was shown to induce apoptosis and cell-cycle arrest, as well as suppress the growth of cancerous cells, in various studies [97,98,99]. A study showed that amlodipine treatment reduced MDA-MB-231 and MCF-7 cell viability in a dose-dependent manner, with IC_50_ values for amlodipine in these cells of 8.66 and 12.60 μM, respectively. The mechanism underlying the cytotoxic effects of amlodipine on the MDA-MB-231 cell line appears to be caspase activation, thus leading to increased caspase-3/7 activity. Amlodipine suppressed the clonogenic proliferation in a dose-dependent manner in MCF-7 cells over a prolonged period of time. On MDA-MB-232 cells, amlodipine demonstrated downregulation of expression in p-ERK1/2 and integrin B1 protein [99]. The anticancer effects of amlodipine were assessed in non-small-cell lung cancer. Amlodipine suppressed the growth of the A549 cell line in a concentration-dependent manner, with an IC_50_ value of 9.641 μM. This inhibition was mediated by the induction of cell-cycle arrest at the G0/G1 phase, without marked apoptosis induction. The molecular mechanism underlying this inhibition was explained by the decrease in phosphorylation levels of PDK1, Akt (both Ser473 and Thr308), mTOR, p70 S6K, and GSK-3β, without changes in total PDK1, Akt, and mTOR levels. Cell migration was also lower in the treated cells compared with the untreated group, exhibiting a concentration-dependent effect. The phosphorylation levels of ERK and c-Raf were reduced in a concentration-dependent manner in amlodipine-treated cells, with no variation detected in total ERK, when compared with untreated cells. Amlodipine significantly suppressed the level of p-EGFR in non-small-cell lung cancer comparatively with the untreated control group, with no change detected in total EGFR [100]. Another study conducted with amlodipine on A431 cells confirmed the results obtained in the previous study [101]. Shiozaki et al. showed that amlodipine could suppress the proliferation of gastric cancer stem cells [102]. The use of amlodipine enhanced the anticancer effect of doxorubicin, inhibiting the proliferation of gastric cancer cells. Furthermore, this combination of amlodipine and doxorubicin could inhibit cell proliferation and spheroid formation in gastric cell lines [103]. Amlodipine could also improve the effect of regorafenib in CCR [104].

### 4.2. Nicardipine

Nicardipine belongs to the DHP class of L-type CCBs mostly used in the treatment of hypertension and angina [178]. Nicardipine increases the expression of Nrf2 and enhances the expression of HO-1. The products of HO-1 subsequently result in inhibition of MMP-9 and cell migration in breast cancer cells [105]. Cotreatment of TNP-470 and nicardipine considerably decreased the growth of the human prostate cancer cell line, PC-3, and inhibited the formation of colonies in a dose-dependent manner [106]. Nicardipine could enhance the cytotoxic effect of temozolomide in glioblastoma stem cells. This cotreatment inhibited autophagy and induce apoptosis [107].

### 4.3. Felodipine

Felodipine is a DHP calcium antagonist indicated for patients with hypertension, chronic stable angina pectoris, and congestive heart failure [179]. Felodipine was capable of decreasing Mz-ChA-1 cell growth in a concentration-dependent manner, with an IC_50_ value of 26 μM. Similar results were observed in the KMCH, CC-LP-1, and TFK-1 cell lines. The results of the study showed an increase in caspase 3/7 following treatment with felodipine, indicating that the cytotoxicity of this agent likely involves the induction of apoptosis [108]. Another study demonstrated a significant reduction in the number of MYO10-induced filopodia in breast cancer cells using this vasodilator. Related results were obtained in pancreatic cancer cells [109]. Filopodia stimulate cell migration in many cell types, and increased filopodia density has been described in cancer, being relevant for cancer progression [110].

### 4.4. Nifedipine

Nifedipine (NIFE) is a DHP L-type calcium channel blocker. This drug inhibits Ca^2+^ influx and is appropriate for the treatment of all types of hypertension [111]. Wu et al. reported that nifedipine suppresses the progression, migration, and immune escape of CRC via a reduction in the expression of NFAT2, inhibiting its nuclear translocation [111]. Lee and colleagues reported that dihydropyridines, such as nifedipine, are able to inhibit the growth of human brain tumor cells [112]. Another study showed that application of 10 μM or 100 μM of nifedipine for 24 h significantly decreased the growth rate of DLD1 cells, but no change in the migratory capacity of these cells was recorded. In these cells, a significant albeit small difference in the S phase of the cell cycle was detected in nifedipine-treated cells compared to untreated cells. In breast cancer cells (MDA-MB-231), treatment with 100 μM nifedipine was able to induce a significant decrease in proliferation and a small decrease in migration [113]. Nifedipine was also shown to potentiate the proapoptotic effect of the chemotherapeutic cisplatin in glioblastoma cells [114].

### 4.5. Diltiazem

Diltiazem, an FDA-approved drug to treat hypertension, is an L-type voltage-gated CCB indicated also for the management of angina and congestive heart failure [180]. One study addressed the antitumor effects of diltiazem, which reduced colony formation, cell migration, and EMT by increasing GDF-15 expression level through inhibiting its proteolytic degradation in different breast cancer cell lines in vitro. Diltiazem administration in vivo also upregulated the serum level of GDF-15, as well as reduced EMT and MMP-9/MMP-2 expression, leading to a decrease in metastasis of breast cancer [115]. Al-malky et al. proved that diltiazem can improve doxorubicin therapy against breast cancer cells (MCF-7 cell line).The results showed that treatment with this vasodilator improved cytotoxic activity, significantly increased the cellular uptake of doxorubicin and aggregation of rhodamine 123, and reduced multidrug resistance [116]. Diltiazem cotreatment, with either 5-FU or gemcitabine, reduced cell viability, induced apoptosis, and caused significant cell-cycle arrest at the S phase in pancreatic cancer cells [181]. Wong et al. proved that diltiazem could induce autophagy in the A549 cell line [117].

### 4.6. Verapamil

Verapamil is a calcium channel blocker that is utilized clinically to treat cardiac arrhythmias, hypertension, and angina [182,183]. Verapamil was demonstrated to promote intracellular drug accumulation when combined with chemotherapeutic agents [118]. This was demonstrated in lung cancer, colorectal, leukemia, carcinoma, and neuroblastoma cell lines [119,120,121,122]. A study confirmed that cotreatment of verapamil with hyperthermia resulted in chromatin fragmentation into nucleosomal oligomers in primary (HT-29) and metastatic (SW620) human colon adenocarcinoma cells, suggesting programmed cell death [123]. The same authors showed that the cotreatment of verapamil with hyperthermia caused a dramatic decrease in cell count [124]. Another study provided evidence that verapamil had an inhibitory effect on G292 osteosarcoma cells. Verapamil could inhibit the activity of G292 cells significantly in the absence of calcium. These results can be reflective of both a decrease in cell viability (measured by membrane integrity) and an increase in cell apoptosis (measured by caspase activity). Furthermore, the role of platelet-derived growth factors was explored in the proliferation and metastasis of osteosarcoma, thus revealing that growth factors can modulate cell migration and matrix metalloprotease production. Moreover, verapamil could also inhibit activity induced by platelet-derived growth factor (PDGF) and insulin-like growth factor (IGF) on this cell line [125]. Chemoresistance in pancreatic cancer appears to result from several mechanisms [126]. Tumors are heterogeneous, comprising a population of cancer stem cells (CSC) that are resistant to cytotoxic drugs and responsible for tumor recurrence. The properties of CSCs that actively cause chemoresistance in cancer cells are not yet fully known, and further study is required [127,128,129]. Chemotherapy-resistant pancreatic cancer cells (L3.6pl and AsPC-1) can be treated with verapamil. This vasodilator could improve the cytotoxic effects of chemotherapeutic drugs and multidrug resistance by targeting the transport function of P-glycoprotein [130]. Verapamil could also induce autophagy in A549 cells [117]. Metabolomics analysis showed that this CCB reversed multidrug resistance in CCR [131].

## 5. Direct Vasodilators

### 5.1. Hydralazine

DNA methylation, histone modification, and noncoding RNA species are the three pillars of epigenetic regulation that can lead to many types of diseases, including different types of cancer [133,184]. These modifications can be inverted by pharmacological agents, such as DNA methylation inhibitors. Hydralazine, a vasodilator and antihypertensive drug, was recently shown to act as a DNA methylation inhibitor by reducing the expression of the DNA methyltransferases DNMT1 and DNMT3a, enzymes responsible for cytosine methylation in mammals [185,186]. An article proved that hydralazine stimulates apoptosis and causes DNA damage in leukemic T cells. T-cell acute leukemia is a type of cancer with a high frequency of mutations in genes encoding for epigenetic regulators. Hydralazine induced apoptosis in a dose-dependent manner in Jurkat, MOLT-4, and CEM-6 cell lines via activation of Bak and loss of mitochondrial membrane potential, followed by production of ROS [133]. Prostate cancer is another type of cancer with frequent epigenetic changes. In another study, DU145, PC-3, LNCaP, and 22Rv1 cell lines were tested with hydralazine and panobinostat alone or as cotreatment to assess their therapeutical potential. Overall, the drugs had a greater cytotoxic effect on the DU145 cell line. Synergy between these two drugs was observed in DU145, PC-3, and LNCaP cell lines. The combined treatment only affected colony formation in PC-3 and DU145, with the clonogenic capacity of the latter cell line being dramatically reduced by exposure to hydralazine alone. When testing the effect of these epidrugs on the invasive and migration capabilities of the cell lines, all cells except LNCaP cells lost their invasive ability following combined treatment. Additionally, in accordance with the invasion assay, DU145 cell’s exposure to hydralazine alone or in combination reduced cell migration [132]. The cotreatment of hydralazine with enzalutamine displayed synergistic effects. This combination in prostate cancer cell lines reduced cell viability, clonogenic and invasive potential, and proliferation, as well as induced DNA damage and apoptosis [134]. Ultrasound hyperthermia can be enhanced by this vasodilator in HCC tumors through modulation of tumor blood flow [135].

### 5.2. Minoxidil

Minoxidil is an ATP-sensitive potassium channel opener and an antihypertensive agent that promotes vasodilation and stimulates hair growth [187,188]. Treatment with minoxidil was shown to decrease cell proliferation and induce proapoptotic effects in ovarian cancer [136]. Qiu et al. showed that, in the breast cancer cell lines MDA-MB-231 and MDA-MB-468, treatment with the vasodilator minoxidil had no effect on cell viability and proliferation whether applied alone or in combination with ranolazine. On the contrary, cell invasion was significantly reduced in a dose-dependent manner by minoxidil and ranolazine, and their combination was additionally effective [137].

## 6. Nitrates

### 6.1. Nitroglycerin

Nitroglycerin (NTG), also called glyceryl trinitrate, is an organic nitrate causing vasodilation via donation of nitric oxide (NO). This vasodilator is used for prophylaxis and treatment of angina pectoris, hypertension, and congestive heart failure, as well as for the induction of surgical hypotension [189]. The interest in the potential use of NTG in cancer treatment arose mainly from studies on the increased vascular permeability exhibited by solid tumors. This phenomenon, possibly related to inflammatory responses to injury or infection [190], was seen by some researchers as a potential mechanism for the targeted delivery of chemotherapeutic agents to solid tumors [138,191]. A study showed that the use of NTG triggers caspase-dependent cell death in human colon carcinoma cell lines (HCT116, SW480, and SW620) [192]. Human PC-3 prostatic adenocarcinoma cells were treated with NTG, resulting in increased survival of these cells [139]. A study on the human leukemia cell line showed that NTG caused a significant decrease in the concentration of cardiolipin (a major mitochondrial lipid), downregulation of respiratory chain complex activities, release of the mitochondrial protein cytochrome *c* into the cytosol, and activation of caspase-9 and caspase-3 [140]. Nagai et al. investigated the effects of NTG on the tumor growth of Lewis lung carcinoma cells in a murine syngraft model. The cotreatment with NTG and PEM significantly reduced tumor growth [141]. Cotreatment therapy with NTG and pemetrexed enhanced the cytotoxic effect and cell growth inhibition in lung cancer [142]. Another study proved that NTG could decrease the expression of TS in a dose-dependent manner in A549 and H1703 cell lines. Moreover, NTG could also enhance the cytotoxic effect and growth inhibition of cisplatin [193].

### 6.2. Isosorbide Mononitrate

Isosorbide mononitrate (ISMN), an organic nitrate approved by FDA in 1997, is used for the treatment of cardiovascular disorders, comprising pectoris, acute myocardial infarction, angina, and congestive heart failure. ISMN is a prodrug that can be activated through in vivo metabolization into NO, a potent gas that produces vasodilation via the eNos/NO/cGMP pathway [194,195]. A study conducted by Wang et al. showed that cotreatment of aspirin and ISMN had a synergistic inhibitory effect on HCT116 and SW620 cell lines. ISMN treatment alone had a minimal effect on cell growth and proliferation. In addition, this cotreatment improved the apoptosis-inducing effect on HCT116 cells, through changes in nuclear morphology, phosphatidylserine translocation, caspase-3 activation, and poly(ADP-ribose) polymerase (PARP) cleavage. A significantly larger amount of chromatin condensation was also shown, along with an elevated apoptotic rate and increased caspase-3 activity [143]. Furthermore, ISMN was shown to inhibit angiogenesis, tumor growth, and metastasis in a chick model of the chorioallantoic membrane (CAM) and a mouse model of Lewis lung carcinoma (LLC) [144]. In another study, codelivery of artesunate and ISMN in ovarian cancer cells (SKOV3 and HO8910) was investigated. The results revealed that the cotreatment could induce the production of ROS, contributing to mitochondrial damage. Additionally, DNA damage and cell-cycle arrest at the G0/G1 phase leading to apoptosis was verified [145].

### 6.3. Sodium Nitroprusside (SNP)

SNP is a complex anion that causes vasodilation, and it is used in the management of acute hypertension and in vascular surgery [146,196]. In a study, the effect of TRAIL was assessed in SGC-7901, AGS, MKN45, and MKN28 gastric cancer cell lines. TRAIL was found to be able to inhibit cell growth and induce apoptosis in these cells in a dose-dependent manner. In addition, the authors found that SNP could sensitize these gastric cancer cells to TRAIL-mediated cytotoxicity by stimulating NO release and facilitating apoptosis [146]. Blackburn et al. investigated the possible cytotoxic effects of SNP on U343, U251, and LN-Z308 glioma cell lines. Only U251 and LN-Z308 cell lines demonstrated increased cytotoxicity and decreased development in a dose-dependent manner. In addition, apoptosis was seen in U251 and LN-Z308 cell lines when exposed to low concentrations of 0.5 mM SNP. It was also possible to verify, in these cell lines, nuclear condensation and fragmentation (apoptotic bodies), as well as cytoplasmic shrinkage and blebbing [147].

## 7. Phosphodiesterase 5 (PDE5) Inhibitors

### 7.1. Sildenafil

Sildenafil is an inhibitor of phosphodiesterase type 5 (PDE5), an enzyme that affects cell signaling. Sildenafil competitively binds to PDE5 with cyclic guanosine monophosphate (cGMP) due to their analogous structures, which improves the levels of cGMP to activate protein kinase G, resulting in vasodilation and an increase in blood flow [197]. Overexpression of PDE5 has been detected in multiple types of cancer, including breast cancer, prostate cancer, bladder cancer, colorectal cancer, and lung cancer [54,198,199,200,201,202]. It was found that sildenafil suppressed B-cell chronic lymphocytic leukemia cell growth and induced apoptosis [44]. Sildenafil was shown to enhance the efficacy of doxorubicin by increasing the permeability of the blood–brain–tumor barrier [45]. Moreover, sildenafil increase the efficacy of doxorubicin in breast cancer [47] and prostate cancer cells [46] without toxicity increase. Additionally, codelivery of crizotinib and sildenafil with nanoparticles showed a synergistic effect and enhanced anticancer therapy in the MCF-7 cell line [48], while the combinatorial delivery of sildenafil–crizotinib–palbociclib could increase cancer treatment in the A549 cell line [49]. Sildenafil also increased the effects of other chemotherapeutic agents such as mitomycin C, doxorubicin, cisplatin, and gemcitabine in pancreatic and bladder cancer cells [50]. Roberts et al. stated that this vasodilator could improve the cytotoxicity of celecoxib, a nonsteroidal anti-inflammatory drug, in tumor cells, including colorectal cancer, hepatoma, glioblastoma, and medulloblastoma, via activation of CD95-induced cell death [51]. A study showed that sildenafil induced cell-cycle arrest at the G1 phase and apoptosis with the increasing accumulation of intracellular ROS in a concentration- and time-dependent manner in human colorectal cancer cell lines (HT-29, SW489, SW629, HCT116, and SW116) [52]. Another study confirmed that sildenafil could reduce the cell viability, number of colonies, and ability of HeLa and SiHa cells to migrate and invade in a dose- and time-dependent manner [53]. Sildenafil was also tested against RD and RH30 rhabdomyosarcoma cell lines. The results showed that this vasodilator decreased cell migration and viability in a dose-dependent manner [55].

### 7.2. Tadalafil

Tadalafil was approved by the FD in 1998, and it is prescribed and used in the therapy of erectile dysfunction [57]. The cotreatment with tadalafil and green tea in prostate cancer was shown to reduce cell proliferation [58]. Similary, tadalafil enhanced the anticancer effect of cisplatin with increased apoptosis in the PC-3 cell line [59]. Tuttle et al. demonstrated that this vasodilator could decrease the viability of UM1, UM47, UM6, and CAL27 cell lines [203]. Cotreatment of tadalafil and cisplatin showed efficiency in the treatment of non-small-cell lung cancer (A549 and SK-MES-1) [56].

## 8. Calcium Sensitizers

### Levosimendan

Levosimendan is a calcium sensitizer that has vasodilatory effects through the stimulation of adenosine triphosphate-dependent potassium channels used for the management of hypertension and heart failure [204,205]. Levosimendan has been applied to numerous tumor cell lines across 19 different types of cancer. The cancer types sensitive to levosimendan include stomach, endocrine, renal, colorectal, bladder, osteosarcoma, melanoma, prostate, and sarcoma, with the most sensitive being hematopoietic lymphoma. Notably, the EC_50_, IC_50_, and GI_50_ values for the SU-DHL-8 cell line were 0.604 μM, 0.604 μM, and 0.512 μM, respectively. According to this study, it was possible to understand that the antitumor activity of levosimendan mainly originates from the modulation of the RNA processing pathway via the inhibition of atypical kinase RIOK1 [96].

## 9. Critical Appraisal of the Use of Vasodilators in Cancer in the Future

Currently, cancer represents the major public health problem and the second cause of death worldwide [206,207]. Although there is no standard treatment scheme adopted, most patients are generally submitted to the primary treatments: radiotherapy, chemotherapy, and surgery [208]. These treatments, although relatively effective, have associated side-effects that can reduce the quality of life of the patient and, in some cases, lead to interruption of treatment [209]. In this way, the use of repurposed drugs, such as vasodilators, can improve the therapeutic ratio of already existing treatments, thus combining their advantages while reducing the associated side-effects. Several in vitro and preclinical data supporting the use of vasodilators against cancer have been carried out, with some but little evidence provided by clinical trials to validate preclinical studies (Table 2).

Since ACEs and ARBs share a main mechanism of action, which is to act as renin–angiotensin–aldosterone system (RAAS) antagonists, they inhibit angiogenesis and reduce the induction of cancer growth, which may decrease cancer risk over time [210]. Other possible mechanisms of action of ACE inhibitors such as enalapril, captopril, and perindopril against tumor cells may involve the inhibition of matrix metalloprotease activity, reduction in vascular endothelial growth factor expression, and interference with the RAAS system [27]. For CCBs, a possible mechanism of action for the anticancer effect could be related to the fact that these vasodilators may restore the dysregulation of Ca^2+^ homeostasis, which has been implicated in the development and progression of cancer [211]. NO is a molecule that affects numerous critical functions in the body. Therefore, in cancer therapy, the application of nitrates is helpful as chemo- and radiotherapeutic sensitizing agents. Malignancies are characterized by hypoxia, which stimulates pathways preparing cancer cells for survival against cell death mechanisms including DNA damage, autophagy, and apoptosis. NO donors, by increasing tumor perfusion, can reverse this effect [212,213]. In the last decade, an increased expression of PDE5 in various human cancers has been reported [214,215,216,217]. Accordingly, PDE5 inhibitors can be a potent anticancer drug. The mechanism of action underlying the anticancer effect of this vasodilator can be explained by the activation of signaling pathways, mostly the PKG, which can inhibit growth, as well as induce apoptosis and autophagy [218]. The mechanism of action against cancer underlying the effects of direct vasodilators is not yet fully understood. One way in which these drugs could be repurposed is through the use of synergistic combination models, which have been described throughout the manuscript as potential treatments for cancer therapy. Synergistic combinations of drugs generally overcome toxicity and other side-effects associated with the administration of high doses of single drugs [219].

## 10. Conclusions

**Figure 2:**
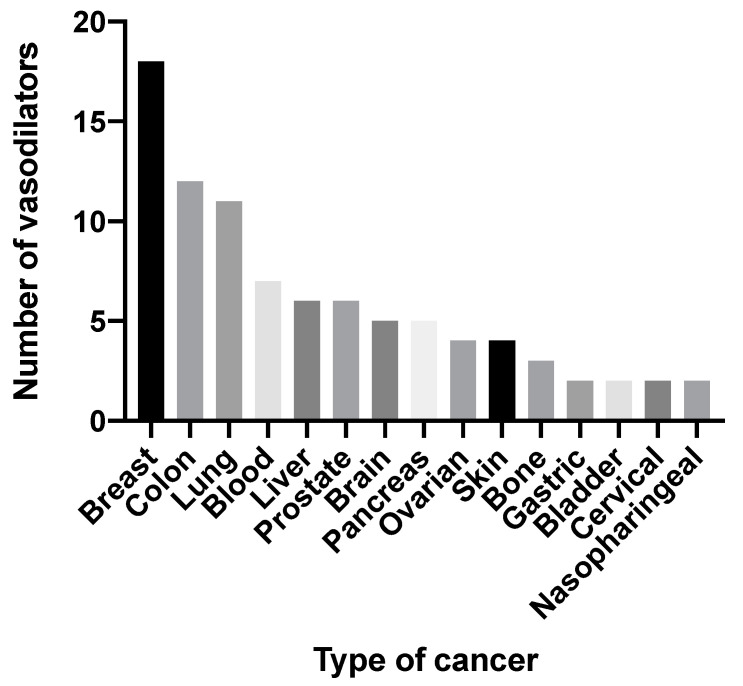
Summary of data evidence of anticancer activity of vasodilator drugs by cancer type, as reported in this review.

The use of vasodilators as a treatment for cancer is a promising area of research that has the potential to improve outcomes for patients with cancer. These drugs can have both protumor and antitumor effects depending on the specific drug and cancer type being studied. On one hand, they can increase blood flow to the tumor, which can promote its growth and proliferation. On the other hand, they can inhibit the formation of new blood vessels in tumors, which can stop tumor growth. Additionally, vasodilators can enhance the effectiveness of antineoplastic drugs already used for cancer therapy. Drug repurposing is a promising approach in the fight against cancer, and vasodilators have shown potential as repurposed drugs for treating various types of cancer (Figure 2), by increasing blood flow to tumors and impacting cancer cell growth. However, more research is needed to fully understand the effects of vasodilators on cancer cell lines and to determine their potential as a treatment for cancer. It is important to note that the effects of vasodilators on cancer cells in a laboratory setting may not necessarily translate to the same effects in humans. Therefore, further research is necessary to fully understand the potential of vasodilators as a therapeutic strategy for cancer patients, such as clinical trials, since only 11 of the 24 vasodilators mentioned on this study have reached clinical trials as anticancer agents.

## Figures and Tables

**Table 1:** Vasodilator drugs against different types of cancer. The table shows only the vasodilator drugs tested on tumor cell lines.

Drug	Drug Class	Clinical Use	Cell Lines	Effects In Vitro	References
Enalapril	ACE inhibitor	Hypertension and heart failure	HCT116, SW620, CT26, HT29, SW40, and HL60	Inhibits cell proliferation, suppresses EMT, and induces apoptosis	[26,27,28,29,30,31,32]
Captopril	ACE inhibitor	Hypertension and congestive heart failure	DU145, HCT116, and Hs578T	Inhibits mitosis, growth, and migration	[33,34,35,36,37,38,39,40,41,42]
Trandolapril	ACE inhibitor	Hypertension, congestive heart failure, and after myocardial infarction	K562, KU812, U937, and HL60	Inhibits cell growth and induces apoptosis	[43]
Sildenafil	PDE5 inhibitor	Erectile dysfunction and pulmonary hypertension	HT-29, SW489, SW620, HCT116, SW116, PC-3, DU145, 4T1, MCF-7, A549, HeLa, SiHa, RD, and RH30	Inhibits proliferation, migration, and invasion; induces apoptosis, cell-cycle arrest at G1 phase, and accumulation of ROS	[44,45,46,47,48,49,50,51,52,53,54,55]
Tadalafil	PDE5 inhibitor	Erectile dysfunction	PC-3, UM1, UM47, UM6, CAL27, A549, and SK-MES-1	Inhibits proliferation	[56,57,58,59]
Azilsartan	ARB	Hypertension	HepG2, A549, MCF-7, and MDA-MB-231	Induces oxidative stress, cytochrome c release, cytotoxicity, cell-cycle arrest, and suppression of NF-kB/IL-6/JAK2/STAT3 signaling pathway	[60,61,62]
Candesartan	ARB	Congestive heart failure and hypertension	CT-26, SW480, HOS, MG63, and U-2OS	Induces E-cadherin, downregulation of MMP3/9, inhibits Wnt/β-catenin signaling, and induces apoptosis	[63,64,65,66,67,68]
Irbesartan	ARB	Hypertension	HCCLM3, HMHCC97-H, HMHCC97-L, SMMC-7721, Huh-7, Hep-3B, PLC, MCF-7, T47D, ZR-75-30, MDA-MB-231, MDA-MB-435, and MDA-MB-468	Inhibits adhesion of HCC cells to endothelial cells and suppresses cell proliferation	[69,70]
Losertan	ARB	Hypertension	MCF-7, CT-26, Huh-7, JHH-6, MHCC97H, HepG-2, and SMMC-7721	Induces apoptosis and G1 cell-cycle arrest	[71,72,73,74,75,76,77,78]
Olmesartan	ARB	Hypertension	A549, HeLa, MCF-7, Me 4405, Sk-Mel-28, PC-3, Du145, MDA-MB-468, and HEK	Inhibits growth; induces apoptosis and ROS	[79,80,81,82,83,84,85]
Telmisartan	ARB	Hypertension	GIST-T1, PC-3, MDA-MB-468, and DU145	Induces cell-cycle arrest in the G0/G1 phase and apoptosis; inhibits cell proliferation	[86,87,88,89,90,91,92,93,94]
Valsartan	ARB	Hypertension	CNE-2	Inhibits growth	[95]
Levosimendan	Calcium sensitiser	Acute and advanced heart failure and hypertension	SU-DHL-8	Inhibits RIOK1	[96]
Amlodipine	CCB	Hypertension, angina and coronary artery disease	MDA-MB-231, MCF-7, A549, and A431	Inhibits proliferation, invasion, colony formation, and cell-cycle arrest at G0/G1 phase	[97,98,99,100,101,102,103,104]
Nicardipine	CCB	Hypertension and angina	PC-3, 4T1, JC, and MDA-MB-231	Inhibits cell migration and colony formation and increases Nrf2 expression	[105,106,107]
Felodipine	CCB	Hypertension, chronic stable angina pectoris and congestive heart failure	Mz-ChA-1, KMCH, CC-LP-1, and TFK-1	Increases caspase 3/7 and decreases cell viability	[108,109,110]
Nifedipine	CCB	Hypertension	DLD1 and MDA-MB-231	Suppresses cancer progression, migration, and immune escape; reduces expression of NFAT2; induces cell-cycle arrest at S phase	[111,112,113,114]
Diltiazem	CCB	Hypertension, angina, and congestive heart failure	MCF-7, JC, 4T1, MDA-MB-231, and A549	Attenuates colony formation, cell migration, and EMT	[115,116,117]
Verapamil	CCB	Hypertension and angina	HT-29, G292, L3.6pl, AsPC-1, G-UVW, G-CCM, G-MCF, P388, and A549	Promotes intracellular drug accumulation, fragmentation of chromatin; decreases cell viability and migration; increases cell apoptosis	[117,118,119,120,121,122,123,124,125,126,127,128,129,130,131]
Hydralazine	Direct vasodilator	Hypertension	Jurkat, MOLT-4, CEM-6, DU145, LNCaP, 22Rv1, PC-3, HeLa, CaSki, SiHa, A549, and H1703	Loss of mitochondrial membrane, ROS production, and reduced colony formation, invasion, and migration capabilities	[132,133,134,135]
Minoxidil	Direct vasodilator	Hypertension	PC-3, LNCaP, LaPC4, HepG2, MDA-MB-231, MDA-MB-468, and OVCAR-8	Increases ROS accumulation; induces apoptosis	[136,137]
Nitroglycerin	Nitrate	Angina pectoris, hypertension, congestive heart failure, and for induction of surgical hypotension	HCT116, SW480, SW620, and PC-3	Decreases concentration of cardiolipin, downregulates respiratory chain complex activities, releases cytochrome *c* into the cytosol, and activates caspase-9 and caspase-3	[138,139,140,141,142]
Isosorbide minitrate	Nitrate	Angina pectoris, acute myocardial infraction, and congestive heart failure	HCT116, SW620, SKOV3, and HO8910	Inhibits cell growth and proliferation; induces apoptosis, chromatin condensation, ROS production, and mitochondrial damage	[143,144,145]
Sodium Nitroprusside	Nitrate	Acute hypertension and vascular surgery	SGC-7901, AGS, MKN45, MKN28, U343, U251, and LN-Z308	Apoptosis induction and cell growth inhibition	[146,147]

ACE: angiotensin-converting enzyme; ARB: angiotensin receptor blockers; CCB: calcium channel blockers; EMT: epithelial-to-mesenchymal transition; ROS: reactive oxygen species; HCC: hepatocellular carcinoma.

**Table 2:** Clinical trials involving vasodilators against types of cancer.

Vasodilator	Type of Cancer	Clinical Trial	Status	Identifier Trial Number (https://clinicaltrials.gov) accessed on 7 February 2023
Enalapril	Woman breast	Not applicable	Completed (2015)	NCT00895414
Captopril	Lung	Phase 2	Completed (2016)	NCT00077064
Perindopril	Colorectal	Phase 2	Completed (2018)	NCT02651415
Losartan	Pancreatic Pancreatic Breast Pancreatic Pancreatic	Phase 2 Phase 2 Phase 2 Phase 2 Phase 1	Active Active Active Recruiting Recruiting	NCT03563248 NCT01821729 NCT05097248 NCT05077800 NCT05365893
Amlodipine	Breast Breast	Phases 1 and 2 Phase 2	Completed (2021) Recruiting	NCT02834403 NCT05660083
Verapamil	Brain Lymphoma	Phase 2 Phase 1	Completed (2017) Active	NCT00706810 NCT03013933
Hydralazine	Breast Ovarian Lung Rectal Cervical Breast Refractory solid tumors Cervical	Phases 1 and 2 Phase 3 Phase 1 Phases 1 and 2 Phase 3 Phase 2 Phase 2 Phase 3	Withdrawn Completed (2009) Completed (2013) Withdrawn Completed (2010) Completed (2006) Completed (2006) Completed (2018)	NCT00575978 NCT00533299 NCT00996060 NCT00575640 NCT00532818 NCT00395655 NCT00404508 NCT02446652
Minoxidil	Ovarian	Phase 2	Recruiting	NCT05272462
Nitroglycerin	Rectal Lung Prostate Lung Lung Brain	Phase 1 Phase 2 Phase 3 Phase 2 Phase 2 Phase 2	Completed (2021) Completed (2017) Completed (2013) Completed (2020) Unknown (2009) Completed (2020)	NCT01407107 NCT01210378 NCT01704274 NCT01171170 NCT00886405 NCT04338867
Sildenafil	Lung Solid tumor	Phases 2 and 3 Phase 1	Completed (2011) Completed (2019)	NCT00752115 NCT02466802
Tadalafil	Pancreatic Liver, pancreatic, and colorectal Head and neck Head and neck Pancreatic Gastric Pancreatic Myeloma Head and neck	Phase 2 Phase 2 Phases 1 and 2 Phase 2 Phase 1 Phase 2 Phase1 Phase 2 Phase 2	Recruiting Completed (2022) Completed (2021) Active Completed (2018) Not yet recruiting Completed (2018) Completed (2014) Completed (2014)	NCT05014776 NCT03785210 NCT02544880 NCT03993353 NCT01903083 NCT05709574 NCT01342224 NCT01374217 NCT01697800

## Data Availability

Not applicable.
